# High Temperature Tolerance in a Novel, High-Quality *Phaseolus vulgaris* Breeding Line Is Due to Maintenance of Pollen Viability and Successful Germination on the Stigma

**DOI:** 10.3390/plants12132491

**Published:** 2023-06-29

**Authors:** Teresa Rose, Claudia Lowe, Javier A. Miret, Hannah Walpole, Kirstie Halsey, Eudri Venter, Milan O. Urban, Hector Fabio Buendia, Smita Kurup, Donal Martin O’Sullivan, Steve Beebe, Sigrid Heuer

**Affiliations:** 1 Rothamsted Research, Harpenden AL5 2JQ, UK; tess.rose@rothamsted.ac.uk (T.R.); c.lowe@kew.org (C.L.); kirstie.halsey@rothamsted.ac.uk (K.H.); smita.kurup@rothamsted.ac.uk (S.K.); 2 Department of Crop Science, University of Reading, Whiteknights P.O. Box 217, Reading, Berkshire RG6 6AH, UK; javier.miret-barrio@rothamsted.ac.uk (J.A.M.); d.m.osullivan@reading.ac.uk (D.M.O.); 3 Centro Internacional de Agricultura Tropical (CIAT), Kilometro 17, Recta Cali-Palmira, Apartado Aereo, Cali 6713, Colombia; m.urban@cgiar.org (M.O.U.); s.beebe@cgiar.org (S.B.); 4 National Institute of Agricultural Botany (NIAB), Lawrence Weaver Road, Cambridge CB3 0LE, UK

**Keywords:** heat tolerance, common bean, pollen structure

## Abstract

The common bean (*Phaseolus vulgaris* L.) is an important nutritional source globally but is sensitive to high temperatures and thus particularly vulnerable to climate change. Derived from a breeding program at CIAT (Colombia), a heat-tolerant breeding line, named heat-tolerant Andean-type 4 (HTA4), was developed by a series of crosses of parents with a small-bean tepary genotype (*Phaseolus acutifolius* L.) in their pedigree, which might be the donor of heat stress (HS) tolerance. Importantly, in HTA4, the large, commercially desirable Andean-type beans was restored. To assess underlying tolerance mechanisms, HTA4, together with a heat-sensitive Colombian variety (Calima), was exposed to HS (31 °C/24 °C HS vs. 26 °C/19 °C day/night) under controlled environment conditions. Vegetative growth and photosynthetic performance were not negatively impacted by HS in either genotype, although senescence was delayed in Calima. HS during the reproductive stage caused an increase in pod number in Calima but with few fully developed seeds and many pods aborted and/or abscised. In contrast, HTA4 maintained a similar filled pod number under HS and a higher seed weight per plant. Pollen showed high sterility in Calima, with many non-viable pollen grains (24.9% viability compared to 98.4% in control) with a thicker exine and fewer starch granules under HS. Calima pollen failed to adhere to the stigma and germinate under HS. In HTA4, pollen viability was significantly higher than in Calima (71.1% viability compared to 95.4% under control), and pollen successfully germinated and formed pollen tubes in the style under HS. It is concluded that HTA4 is heat tolerant and maintains a high level of reproductive output due to its ability to produce healthy pollen that is able to adhere to the stigma.

## 1. Introduction

In 2022, the global mean temperature was estimated to be 1.15 °C above the 1850–1900 pre-industrial average [1]. Some areas are already experiencing extreme weather events, such as droughts, floods, and heat waves. Both chronic heat and acute heat shock have been shown to impact plant performance and reduce yield with each degree of warming in some of the world’s most economically important crops, including maize (−7.4%), wheat (−6.0%), rice (−3.2%) and soybean (−3.1%; [2]).

The common bean (*Phaseolus vulgaris* L.), harvested either before physiological maturity as green pods, or as mature dry beans, is an important source of dietary protein, complex carbohydrates, and micronutrients in many areas globally. Around 27.5 million tonnes of dry beans are produced annually, over an area of 34.8 million hectares globally [3]. Common beans are mainly consumed in countries, where they are produced and are cultivated by smallholders, and on an industrial scale. Countries with high rates of bean consumption per capita (mostly in Central and South America, the Caribbean, East Africa and some Asian regions) also import beans, to meet demand. The Americas and Asia are the most important producing regions [3].

It has been established that heat stress (HS) can alter flowering time, cause asynchrony between male and female reproductive development, and trigger the abortion of buds, flowers and seeds, all of which lead to reduced yield [4,5]. Both chronic and developmental stage-specific HS can reduce fertilization, and therefore seed set [6,7,8] by damaging the sensitive reproductive organs and developing gametes. Male gametophytic development is particularly sensitive to heat in a wide range of crop plants, including wheat [9,10], cotton [11], tomato [12] and beans [4,13], with the development of the meiocytes and microspores being disproportionately affected [14,15,16]. Pollen sterility and anther indehiscence are common in HS plants [5,13]. It has been reported that while pollen becomes less sensitive to heat as it matures, the stigma becomes more sensitive towards anthesis [17]. Such effects have been observed in the common bean at temperatures exceeding 30 °C during the day, and 20 °C at night [18]. These effects were previously attributed to an imbalance in sugar homeostasis and damage to the membranes of the tapetum and developing pollen grains [19].

Bean crops are negatively impacted globally by adverse weather conditions, and one of the goals of the CIAT (Cali, Colombia) extensive breeding program is to develop heat-tolerant breeding lines. Modern *Phaseolus vulgaris* derive from two gene pools of Andean and Mesoamerican origin [20]. While Mesoamerican varieties are generally more tolerant to various environmental stresses, they produce smaller beans that are less popular with consumers. Heat-tolerant Andean-type 4 (HTA4) is a promising new genotype which was developed at CIAT using three accessions with a complex pedigree, including a small-beaned tepary genotype (*Phaseolus acutifolius* L.; G40020, GID 284). Tepary bean germplasm contains heat tolerance [21], and HTA4, whilst producing the desirable, large, Andean bean type, has displayed heat tolerance under field conditions (CIAT).

Here, we aimed to confirm the heat tolerance of HTA4 in controlled cabinet experiments, and move towards elucidating the mechanism(s) of tolerance in this genotype. To achieve this, we measured a range of phenological and physiological factors and contrasted the results with the popular heat-sensitive Colombian Andean type, Calima.

## 2. Results

### 2.1. Vegetative Traits

#### 2.1.1. Plant Growth and Development

**Figure 1:**
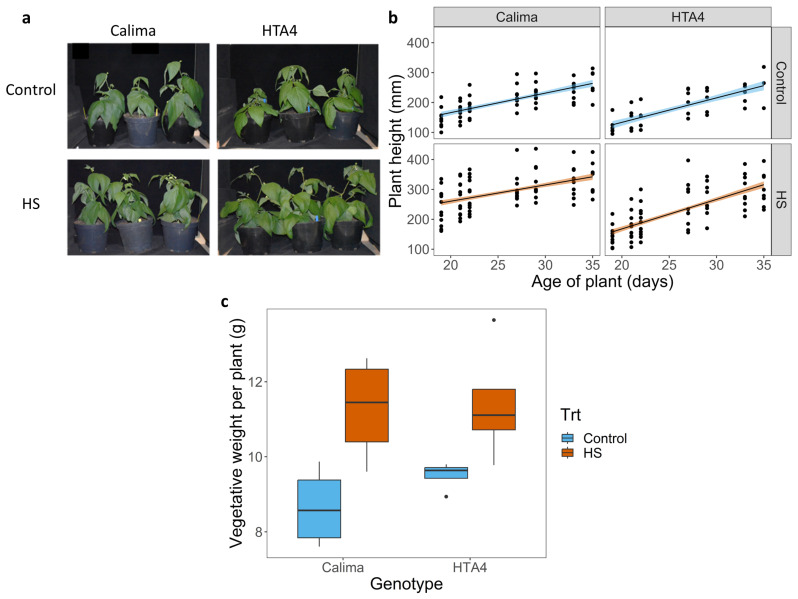
(**a**) *Phaseolus vulgaris* cv. Calima and HTA4 at flowering, under control (day 31) and HS (day 33) conditions. (**b**) Fitted linear regression of plant height (mm) over plant age in days. Solid lines show the fitted model with shading around the line showing standard error of the predicted model means. There was a significant interaction between plant age and genotype (*p* < 0.01), and between genotype and treatment (*p* < 0.01). (**c**) Boxplot of total vegetative dry weight per plant, summarized by genotype (Calima and HTA4) and temperature treatment (blue fill = Control, orange fill = HS). In a linear regression of vegetative dry weight predicted by genotype, treatment, and the interaction between them, treatment was the only significant variable (*p* < 0.01).

Under chronic heat stress at 31 °C/24 °C day/night, vegetative growth was not affected for either genotype, compared to plants grown under control conditions of 26 °C/19 °C (Figure 1a). In the intolerant genotype Calima, plant height increased more rapidly under HS, averaging 250 mm at day 20, compared to 150 mm under control conditions (Figure 1b). HS Calima plants remained taller than their control counterparts throughout their life cycle. HTA4 did not show such a rapid initial height increase, but heat-treated HTA4 plants were significantly taller than the control plants (300 mm vs. 250 mm, respectively) by day 35, when measurements ceased (Figure 1b, Table A1). Total vegetative dry weight (DW) increased significantly for both cultivars under heat stress (Figure 1c, Table A2). DW increased by 13% in HTA4 (from 9.75 g to 11 g) and by 35% (from 8.5 g to 11.5 g) in Calima. Both cultivars switched to reproductive growth around 2 days earlier under heat stress compared to control conditions.

#### 2.1.2. Chlorophyll Density, Leaf Senescence, and Quantum Efficiency of PSII

**Figure 2:**
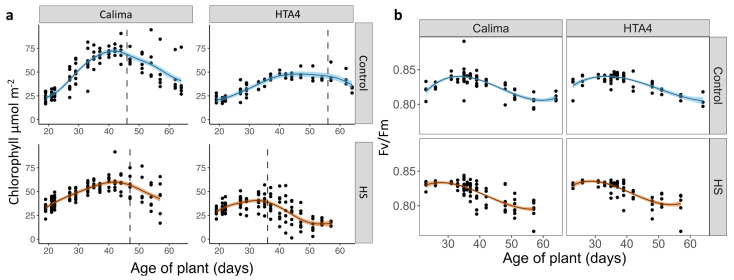
(**a**) Fitted generalized additive model of chlorophyll density (µmol m${}^{-2}$) in the first trifoliate leaf over plant age in days with solid lines for model predictions and shading to show the standard error of the predicted model means. (**b**) Fitted polynomial linear regression of Fv/Fm in the first trifolate leaf over plant age in days. Solid lines show fitted model predictions and shading to show the standard error of the predicted means. Genotype and treatment are significant predictors in the model (*p* = 0.03, *p* < 0.01, respectively) of the interaction terms. Only treatment interacted significantly with the plant age (*p* < 0.01).

The estimated maximum chlorophyll density (µmol m${}^{-2}$) was higher in Calima compared to HTA4 but decreased under heat stress in both Calima (hlheat: 59.97 µmol m${}^{-2}$; control: 72.21 µmol m${}^{-2}$) and HTA4 (heat: 40.55 µmol m${}^{-2}$; control: 47.97 µmol m${}^{-2}$; Figure 2a). A decrease in chlorophyll density, indicating the start of leaf senescence, was observed earlier under HS (36d) compared to control conditions (56d) in HTA4, but not in Calima (47/46 d heat/control; Figure 2a).

Fv/Fm, representing chlorophyll fluorescence-related photosynthetic efficiency, was significantly different over time under the two treatments but not between genotypes, demonstrated by a significant interaction between plant age and treatment (Figure 2b, Table A3). Initial Fv/Fm was, on average, higher under heat stress compared to control conditions, as the predicted intercept was significantly different for both genotype and treatment. However, a lack of significant interaction between genotype and treatment intercepts shows that the response of Fv/Fm to heat did not differ between the genotypes.

### 2.2. Reproductive Traits

#### 2.2.1. Pod and Seed Characteristics

**Figure 3:**
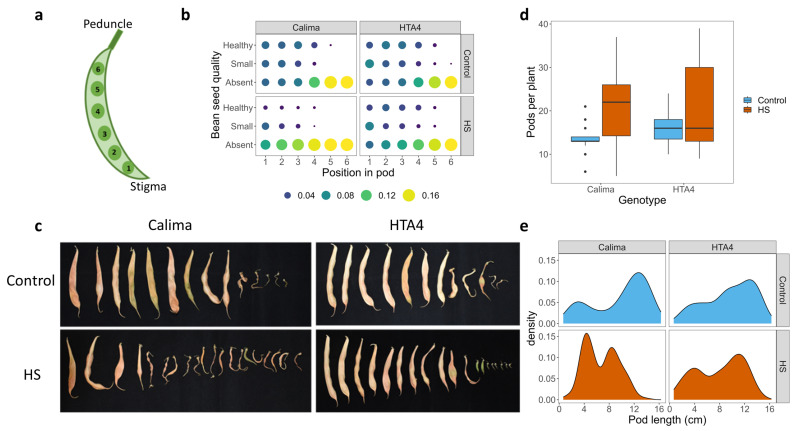
(**a**) Diagram of seed location within the pod. Position in pod starts from 1 at the distal end, to 6 and the proximal end. (**b**) Quality and proportion of bean seeds within pod. Size and color of points represent the proportion of seeds from the respective treatment and genotype of the given quality and position in pod. (**c**) Photographs of typical control and heat-stressed pods at final maturity. (**d**) Boxplot showing the number of pods per plant for the two genotypes (Calima and HTA4) under two temperatures (control and HS). In a generalized linear regression with numbers of pods per plant predicted by genotype, treatment, and the interaction between them, treatment was the only significant predictor (*p* < 0.01). (**e**) Smoothed histogram showing the length of pods from the two genotypes (Calima and HTA4) under two temperatures (control and HS).

The quality and position of seeds within the pods remained proportionally similar for HTA4 under HS and control. In contrast, Calima showed a reduction in the number of healthy seeds, as well as overall seed number per pod under HS (Figure 3a,b). All plants had greater success filling the seed positions closest to the stigma/furthest from the peduncle (position 1). HTA4 was able to produce more fully developed seeds under HS compared with Calima and filled up to five seed positions within pods. Representative images of HS and control pods can be seen in Figure 3c.

Under HS, both Calima and HTA4 showed a greater variance in pod number per plant. The mean number of pods per plant increased in Calima (from 13.5 to 21.4) but not in HTA4 (average pod number 15.1; Figure 3d, Table A4) under HS. Pod length under HS was reduced in Calima (Figure 3e), where the most abundant pod length fell from approximately 12 cm in control plants to a maximum of approximately 4 cm under HS. It is important to note, however, that we often observed pods less than 1 cm which had fallen from heat-treated experimental plants, and although these were collected and stored, it is not inconceivable that some were missed. Therefore, the data for final pod numbers and average pod lengths do not include pods that were excised at a very early stage and, as a result, were missed from the respective counts. As pod excision was higher under HS, it could be that the total pod numbers of Calima and HTA4 were higher under HS than reported in our data. In HTA4, the most common pod length decreased from around 14 cm to 11 cm (control and HS, respectively), reflecting the reduction of seeds in the sixth pod position, but overall, the larger pods remained more abundant than those aborted at an early developmental stage.

**Figure 4:**
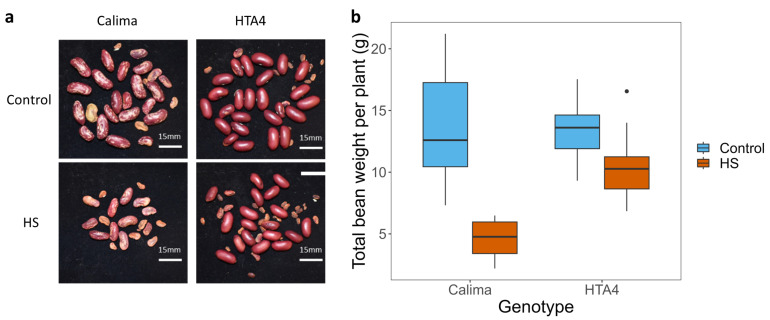
(**a**) Photographs of typical heat-stressed and control seeds. (**b**) Boxplot of total bean seed weight per plant (n = 52), summarized by genotype (Calima and HTA4) and temperature treatment (blue fill = control, orange fill = HS). In a linear regression of bean seed weight predicted by genotype, treatment, and the interaction between them, all were significantly predictive variables (*p* < 0.01).

Beans produced under HS were visibly different from the control beans in both cultivars, being generally misshapen and discolored (Figure 4a). The total bean weight dropped significantly from a mean of 12.5 g to 5 g in Calima under HS, whereas HTA4 maintained a mean total bean weight of 10.5 g under HS, compared to around 13 g under control conditions (Figure 4b, Table A5). Where Calima did produce healthy-looking beans, they were at the end of the pod proximal to the stigma (position 1).

Taken together, these data showed higher reproductive success in HTA4, whilst Calima showed an intolerant response as indicated by an increased number of pods with few fully developed beans under HS.

#### 2.2.2. Anther and Pollen Viability

**Figure 5:**
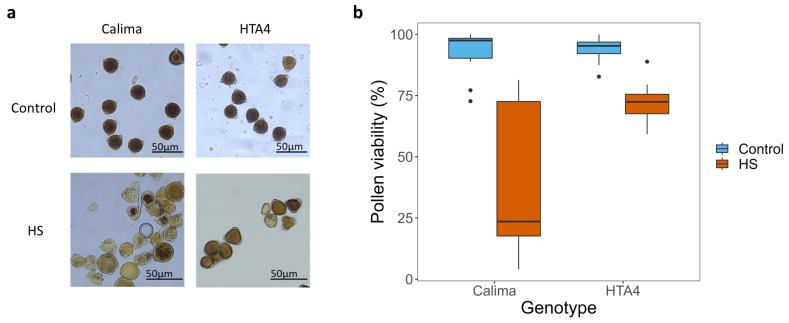
(**a**) Images of Calima and HTA4 pollen under control and HS conditions, pollen were stained with 1:2 iodine:potassium iodide solution and images captured on an Axioimager microscope. (**b**) Boxplot of pollen viability (%), summarized by genotype (Calima and HTA4) and temperature treatment (blue fill = control, orange fill = HS). In a linear regression of pollen viability predicted by genotype, treatment, and the interaction between them, all were significantly predictive variables (*p* < 0.01).

Staining with Lugol’s iodine solution showed a lack of starch in HS Calima pollen, whereas HTA4 had a significantly higher proportion of starch-filled pollen (see Figure 5a for representative images). Starch levels can be used as an indicator of pollen viability because starch biosynthesis is the final step of pollen development [22]. Pollen viability data show that Calima pollen was more severely affected by heat than HTA4 pollen (Figure 5b). Calima pollen viability significantly decreased to 24.9% from 98.4% under control conditions (Figure 5b, Table A6), and the variability between samples was greater. Pollen viability was also reduced in HTA4, but to a lesser extent, from 95.4% to 71.1%, and showed less variability compared to Calima. When assessed microscopically, anthers of HS plants appeared similar to those of control plants and anther dehiscence was unaffected.

**Figure 6:**
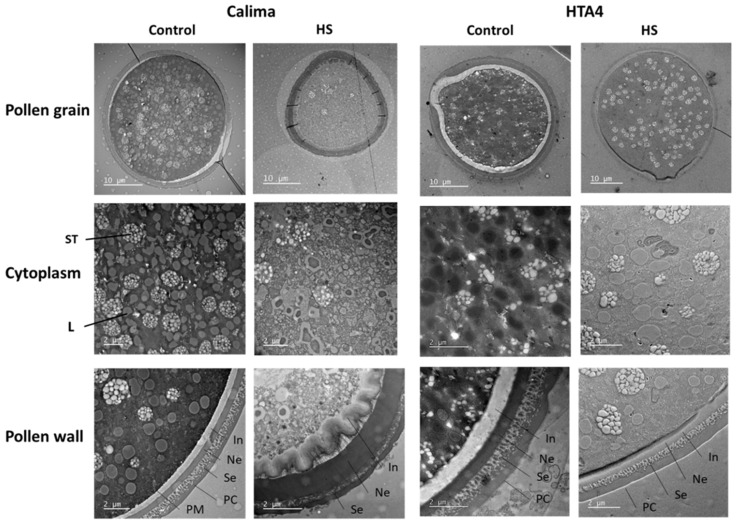
Transmission electron microscope images of Calima and HTA4 pollen under control and HS conditions. Images show whole pollen grain, cytoplasm, and exine. ST, starch; L, lipid body; PC, pollen coat; Se, sexine; In, intine; Ne, nexine; PM, plasma membrane.

Microscopic TEM analysis of pollen grains showed that the fertile-looking pollen (i.e., the 30% classified as viable) of HS Calima plants had a thickened exine (Figure 6), which could hinder pollen rehydration, and/or the formation of pollen tubes from the pores. These pollen grains were also generally smaller compared to controls, and their starch granules appeared structurally different and less abundant. As starch provides energy for pollen tube growth, this would have negative implications for the ability of HS pollen to reach the ovary and fertilize ovules. Pollen of HS HTA4 looked more like control pollen, with a similar exine structure and cytoplasmic constituents.

**Figure 7:**
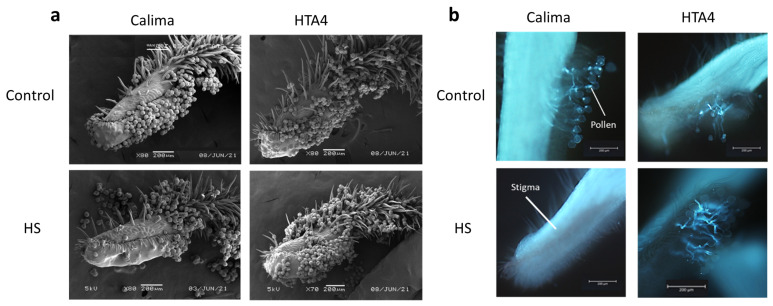
(**a**) Scanning electron micrograph of HTA4 and Calima stigmas, one day post anthesis, under control and HS conditions. (**b**) Aniline blue stained stigmas from HTA4 and Calima plants, grown under control and HS conditions, showing pollen tubes germinating on stigma surface and growing within the style.

SEM analysis showed an absence of pollen on the stigmas of heat-treated Calima plants (Figure 7a). This was because pollen failed to adhere to the stigma and was easily washed off the stigma during sample processing. In contrast, HTA4 pollen germinated and formed pollen tubes in the style under control and HS conditions (Figure 7b).

## 3. Discussion

### 3.1. HTA4 Is Tolerant to HS

As HTA4 is the result of a breeding program that aimed to incorporate the heat tolerance of tepary beans (*Phaseolus acutifolius*) into the Andean race of *Phaseolus vulgaris*, and as HTA4 had exhibited heat tolerance in the chronic stress field and pot trials at CIAT, Colombia (personal communication Steve Beebe, Milan O. Urban), we expected it to be tolerant to the HS applied in this chronic high temperature cabinet experiment. The data presented show that this is indeed the case, and HTA4 not only performed well under HS compared to the control during vegetative development (as measured by plant height and final dry weight), but it also successfully made the switch to reproductive growth, maintained high percentage pollen viability, and fertilized and filled more seeds than its heat-susceptible counterpart. HTA4 switched to reproductive growth two days earlier under HS than the control, and its leaves began to senesce around 20 days earlier than under the control conditions. It is thereby efficiently flowering, setting seed and (re)mobilizing resources to fill the developing seeds, and avoiding prolonged exposure to HS.

Calima, meanwhile, grows rapidly in the vegetative stage, and similarly makes the reproductive switch early, but is unable to translate that into a successful reproductive output. Calima plants continue vegetative growth, do not senesce earlier, and do not mobilize enough resources due to the absence of a strong sink. Where Calima did fill seeds under HS, they were often smaller and/or misshapen and discolored, traits that are actually much more related to their market value than yield. Due to seed/pod failure under HS, Calima continuously produced many pods, the majority of which were aborted or abscised early in development. This early pod abortion and extended reproductive phase has been observed in heat-sensitive *Phaseolus* species previously [5,23].

### 3.2. The Reproductive Stage Is Sensitive to HS

The high rate of pod abortion in Calima under HS, coupled with the negative effect of HS on all pod and seed characteristics measured, indicates that it is the early reproductive stage that is sensitive to HS in Calima, and it is at this stage that HTA4 has a tolerance mechanism. We observed that, where Calima had successfully filled seeds, they were located at the end of the pod proximal to the stigma, suggesting that seed failure might be a consequence of failed fertilization. This phenomenon led us to consider the effects of HS on pollen viability and pollen–stigma interaction.

While the temperature ranges that plants can grow in without suffering heat stress are distinct to each plant species and genotype [24], they are generally narrower for male gametophytic tissues [25]. The morphology of anther tissue was previously shown to be impacted by HS, leading to the decreased or unsuccessful production of pollen grains, and/or anther indehiscence in bean [5] and other crops [26]. Anther indehiscence was not observed under the conditions applied in the current study; however, interestingly, the HT anthers from both cultivars appeared to be morphologically similar to the controls. This indicates that if male sterility causes the differences observed between Calima and HTA4 under HS, pollen viability and stigma receptivity rather than anther indehiscence could be responsible.

Common bean pollen viability has been shown to reduce under HS, particularly when HS is applied during meiocyte and microspore formation [4,13]. Overall, this leads to reduced pollen number, and percentage viability. In this study, pollen viability assays showed a greater drop in pollen viability and an increase in variability between flowers, under HS in Calima compared with HTA4, explaining, to an extent, the reduced reproductive success in this cultivar. In Calima, there was a large proportion of pollen that was small and was lacking cellular contents, including, in some cases, nuclei, indicating that development was arrested at an early stage. Where Calima pollen appeared viable, it showed morphological changes upon closer inspection by TEM, often being smaller and misshapen, with a thicker exine and fewer starch granules. Pollen starch granule size and number decreased in maize under HS [8], and pollen malformations, such as disordered tecta, absent nexine, and uneven surface sporopollenin, were observed in HS rice pollen [27], as well as a thicker exine in HS soybean pollen [28]. Field pea pollen was also shown to be smaller, with reduced interior contents under HS [29]. Non-viable HTA4 pollen was mono-nucleate, while viable pollen appeared structurally similar to control pollen.

High variability in pollen viability within a single anther locule under HS was previously attributed to the amplification of initially small developmental or metabolic differences between microspores by competition for limited nutrients [30], resulting in a mixed population of viable and non-viable pollen within a single anther locule [31]. This could explain the huge variability of data observed in Calima. While the plants in this study have sufficient resources (as photosynthesis and vegetative growth are unaffected or improved), developing pollen could be starved of nutrients because of a problem with nutrient mobilization in the anther, such as loss of integrity of the tapetum, which has been observed under HS previously [28,32]. Soltani et al. [33] previously reported that moderate HS does not negatively affect photosynthesis in a heat-sensitive *Phaseolus vulgaris* cultivar, Redhawk, but HS disrupts the source–sink balance, impacting pollen development and pod filling, with a four-fold reduction in free hexoses in HS beans. Wang et al. [8] showed that the leaf assimilate was remobilized but was not converted to starch in HS maize pollen. Disrupted sugar metabolism was also reported in tomato anthers, leading to reduced pollen viability under HS [16,34].

Although pollen viability drops in HTA4, it is to a lesser extent, and the range is similar to that of the control pollen samples. This demonstrates that HTA4 is able to maintain pollen development under HS and its ability to retain similar levels of variability could be a key trait for tolerance in this genotype.

### 3.3. Calima Pollen Does Not Adhere to the Stigma under HS

Scanning electron microscopy showed an absence of pollen on the stigmas of heat-treated Calima plants, and any pollen present was easily dislodged from the stigma during sample processing. This fits with a previous observation that reduced yield under HS is associated with problems penetrating the stigma [17]. Considering that Calima does have a proportion (c25%) of apparently viable pollen, it might be expected that it could maintain a higher yield than was observed, considering the necessity of only six viable grains to fill each pod. The altered morphology of apparently ‘viable’ HS Calima pollen could explain why this is not the case. The lack of pollen adherence to the stigma might be due to differences in the structure of the exine and/or impaired rehydration as a requirement for germination. So far, it is also unknown if some feedback effect of the pollen grain presence itself affects the stigma receptivity.

Previous studies found morphological and/or biochemical changes in the pistil under HS [35,36], which could be a factor in the failure of pollen to adhere to the stigma surface. Our TEM images, and observation during imaging for pollen viability, show that the stigma appears structurally normal in HS Calima plants but further analysis would be required to rule out the role of stigma damage in the failure of fertilization. However, Monterroso and Wien [4] showed in reciprocal crosses that, in *Phaseolus vulgaris*, applying control pollen to HS stigmas rescued yield to some extent, indicating that the problem is with the pollen and not the stigma, or to a lesser degree due to the stigma. A small pilot study to replicate this in our lab with Calima and HTA4 showed the heat sensitivity of both pollen and stigmas, and plants that had experienced HS to either the male or female reproductive organs showed higher pod abortion rate than controls.

Understanding how HTA4 pollen has avoided or overcome the effects of HS suffered by Calima could be a key to understanding the molecular and physiological mechanism of heat tolerance in common beans.

## 4. Materials and Methods

### 4.1. Plant Material

Calima and HTA4 seeds were provided by Alliance Bioversity International and CIAT (CIAT, Cali, Colombia). HTA4 is a breeding line derived from a cross involving the parents DAA9 × (DAB295 × SAR4). Calima was selected as a control because it is representative of the most important commercial grain class in East Africa and has been released as a variety there. Thus, it is representative of what is currently in the field in production. There are also extensive physiological data on it.

Seeds were sown in 8-inch pots containing custom compost, comprising 75% medium grade peat, 12% screened sterilized loam, 3% medium grade vermiculite, 10% grit (5 mm screened, lime free), 3.5 kg m${}^{-3}$ Osmocote Exact (total N 16%) (supplier: Scotts UK Professional, Ipswich, Suffolk, UK) and 0.5 kg m${}^{-3}$ PG compound fertilizer (supplier: Yara UK Ltd., Harvest House, Europarc, Grimsby, N E Lincolnshire, UK). Plants were kept in Sanyo cabinets under 26 °C/19 °C day/night cycles, with 11 h nights, 7 h days and 3 h ramping periods between the two. All plants were maintained at control temperatures until 7 days post germination to control for germination effects. At this point, plants allocated to the HS condition were transferred to 31 °C/24 °C day/night cycles as specified above. Control plants were transferred to a cabinet, which was maintained at control conditions. Plants were kept well watered and cabinet humidity was maintained at 70% RH. Lights (Phillips T5, 840 W cool white dimmable fluorescent lamps) provided 400 µmol m${}^{2}$ s${}^{-1}$ set at 100 cm height. At the end of pod elongation and the beginning of seed ripening (BBCH stage 80), plants were moved to a glasshouse under ambient conditions for drying until harvest. A total of 60 HTA4 and 60 Calima plants were grown, half under HS and half under control conditions, and plants were allocated either for destructive sampling for imaging or for chlorophyll, yield component and dry weight measurements (plant numbers for each measurement are outlined in the relevant sections below).

### 4.2. Pod and Seed Characteristics

The position and quality of seeds in a total of 922 pods from 24 plants grown in temperate conditions (13 Calima and 11 HTA4) and 28 plants grown in heat stress conditions (14 Calima and 14 HTA4) were measured. Positions in the pod were marked from 1 to 6, starting with 1 at the distal end and going to 6 at the proximal end. Seeds at each position were scored by eye as healthy if the seed was full and without visible defects or discoloration, shriveled if the seed was very small, having a shriveled seed coat or visibly deformed, or absent if no seed was present. Subsequently, the total weight of seeds was measured from each plant. Seed weight was predicted by genotype, treatment and the interaction between genotype and treatment in a linear regression; the significance of the model terms was estimated in an ANOVA framework. All analyses were performed in R (version 3.6.1). For pod counts, all developing pods on each plant were collected; however, in the case that pods were abscised very early, it is possible they were missed from the final count.

### 4.3. Chlorophyll Density

The chlorophyll content of the central leaflet of the first fully emerged trifoliate leaf of 16 plants under temperate conditions (10 Calima and 6 HTA4), and 23 plants under heat stress conditions (12 Calima and 11 HTA4), n = 39, was measured in situ using the optical method with the MC-100 Chlorophyll Concentration Meter (Apogee Instruments Inc., Logan, UT, USA). Measurements were taken in the morning, on two to three days each week, between 19 and 64 days of growth for the control condition plants, and between 19 and 57 days for HS plants. Measurements were stopped earlier in the heat-treated plants, as the oldest leaves had started to drop.

### 4.4. Fv/Fm

The maximum potential quantum efficiency of Photosystem II (Fv/Fm) of the first fully emerged trifoliate leaf was measured in 143 plants under temperate conditions (76 Calima and 67 HTA4) and 147 plants under HS conditions (78 Calima and 69 HTA4), n = 290. Black leaf clips were applied to the leaves 20 min for dark adaption before Fv/Fm was measured in situ, using a Pocket PEA continuous excitation chlorophyll fluorimeter (Hansatech, Norfolk, UK). Measurements were taken regularly in the morning, between 23 and 64 days of growth for the control condition plants and between 23 and 57 days for the HS plants. Measurements were stopped earlier in the heat-treated plants, as the oldest leaves had started to drop. A third-order polynomial regression was fitted with Fv/Fm predicted by plant age in days interacting with both genotype and treatment. The significance of these predictors was estimated using an F test in an ANOVA framework. The model was fitted in R version (3.6.1).

### 4.5. Senescence

Chlorophyll parameters of the maximum chlorophyll density (µmol m${}^{-2}$), plant age at senescence onset, and senescence rate were estimated using generalized additive models (GAMs). Chlorophyll was predicted by a smooth function of plant age (days), with a separate smooth function fitted for each combination of the cultivar and temperature treatment. The optimal number of basis functions was determined to be 9 using REML. The maximum chlorophyll density (µmol m${}^{-2}$) was estimated from the fitted predicted model, the age of senescence onset was calculated as the plant age in days that chlorophyll density fell to 95% of the maximum. The senescence period was calculated as the beginning at senescence onset and ending after 14 days or the end of the experiment, whichever was sooner. The senescence rate was calculated as the change in chlorophyll density over the senescence period. GAMs were fitted in R (version 3.6.1) using the mgcv package (version 1.8-35) [37]).

### 4.6. Plant Height

The plant height was measured using the same plants used for chlorophyll density measurements (Section 2.2). Height in mm from the base to the top of the main stem was measured on seven occasions between 19 and 35 days after planting. Linear regression with plant height predicted by plant age in days interacting with both treatment and genotype was fitted. The significance of each of these predictors was estimated using an F test in an ANOVA framework. The model was fitted in R (version 3.6.1).

### 4.7. Imaging

#### 4.7.1. Pollen Viability

Mature anthers were collected from three flowers from each of four plants from the various treatment conditions described above. Anthers were disrupted on a microscope slide using forceps to release the pollen, which was then stained with Lugol’s iodine (1:2 Iodine:KI solution). Stained pollen was imaged and photographed using a Zeiss Axioimager, and viable pollen was quantified using ImageJ software [38].

#### 4.7.2. TEM of Pollen Grains

Pollen samples were fixed with 4% paraformaldehyde, 2.5% glutaraldehyde in 0.1 M Sorenson’s Phosphate (SP) buffer at pH 7.2 overnight (Ruzin, 2000). They were washed 4× with SP buffer and centrifuged at 35,000 rpm for 5 min. Pollen was embedded in 2% low melting agarose before dehydration through a series of increasing concentrations of ethanol and infiltration with LRWhite resin (AGR1281, Agar Scientific Ltd., Stansted, Essex, UK). Samples were embedded in capsules and polymerized in a 60 °C nitrogen oven overnight. The resin blocks were cut into 100 nm slices and placed onto Pioloform-carbon-coated copper grids for staining with 2.5% uranyl acetate for 20 min [39]. Samples were rinsed with dH_2_O, stained with 3% lead citrate for 3 min and rinsed with dH_2_O again. Imaging was done using a JEOL 2100Plus transmission electron microscope (JEOL, Tokyo, Japan) at 200 kv. A range of representative pollen was imaged, from fertile-looking to non-viable. Particular attention was paid to pollen from heat-stressed plants that appeared viable to explain why it was unable to adhere to the stigma and form pollen tubes.

#### 4.7.3. Cryo-SEM of Pollen on Stigma

Stigmas from three flowers, from each of three individual plants per treatment, were mounted onto the stub using a 1:1 mixture of tissue tek and graphite, earthed with carbon tape to reduce charging. The sample was then rapidly frozen in slushed liquid nitrogen (−207 °C) before being transferred into the Quorum cryo-preparation system. Each sample was etched at −95 °C for 5 min and coated with platinum for 2 min at a current of 5 µA. Cryo-SEM imaging was conducted on the JEOL 6360 LowVac Scanning Electron Microscope (JEOL, Tokyo, Japan) using a 5 kv beam current, spot size 30 and a working distance (WD) of 15 mm.

#### 4.7.4. Pollen Tubes Growing in Style

Newly opened flowers were collected in the morning and fixed in FAA (formalin–acetic acid–alcohol) or 70% ethanol. The pistil was dissected out and washed twice in boiled distilled water, and then placed in a boiling water bath for 1 h. Samples were removed from the water bath and cleared at 60 °C for 1 h with 4 N sodium hydroxide. The samples were washed with dH_2_O, stained with 0.1% Aniline blue in 0.1 M potassium phosphate for 30 min in the dark and mounted on a slide with decolorized Aniline blue. Images were taken with a Zeiss Axioimager fluorescent microscope (Zeiss, Oberkochen, Germany), with 455 nm excitation and 520 nm emission.

## 5. Conclusions

Our data confirm the high temperature tolerance of HTA4 and show that this is related to the maintenance of pollen viability and to the ability of pollen to adhere and germinate on the stigma. This is the first report of a high-quality heat-tolerant *P. vulgaris* genotype with large commercially desirable beans. Genotyping and mapping are ongoing to provide more information for breeders and facilitate the development of heat-tolerant bean cultivars.

## Data Availability

The data presented in this study are available on request from the authors.

## Appendix A

**Table A1:** Plant height ANOVA table. Significance (Sig.) indicated with stars; *** *p* value between 0 and 0.001, ** *p* value between 0.001 and 0.01, no stars *p* value > 0.1.

	Df	Sum Sq	Mean Sq	F Value	Pr (>F)	Sig.
Plant_age_days	1	410,114.31	410,114.31	180.11	0.0000	***
Genotype	1	103,714.67	103,714.67	45.55	0.0000	***
Treatment	1	291,560.44	291,560.44	128.05	0.0000	***
Plant_age_days:Genotype	1	20,509.77	20,509.77	9.01	0.0030	**
Plant_age_days:Treatment	1	14.12	14.12	0.01	0.9373	
Genotype:Treatment	1	27,996.44	27,996.44	12.30	0.0005	***
Plant_age_days:Genotype:Treatment	1	3214.95	3214.95	1.41	0.2359	
Residuals	240	546,481.82	2277.01			

**Table A2:** Vegetative dry weight ANOVA table. Significance (Sig.) indicated with stars; ** *p* value between 0.001 and 0.01, no stars *p* value > 0.1.

	Df	Sum Sq	Mean Sq	F Value	Pr (>F)	Sig.
Genotype	1	0.95	0.95	0.65	0.4372	
Treatment	1	20.63	20.63	13.98	0.0028	**
Genotype:Treatment	1	0.52	0.52	0.35	0.5645	
Residuals	12	17.70	1.48			

**Table A3:** Fv/Fm ANOVA table. Significance (Sig.) indicated with stars; *** *p* value between 0 and 0.001, * *p* value between 0.01 and 0.05, no stars *p* value > 0.1.

	Df	Sum Sq	Mean Sq	F Value	Pr (>F)	Sig.
poly(Plant_age_days, 3)	3	0.04	0.01	122.40	0.0000	***
Genotype	1	0.00	0.00	5.08	0.0250	*
Treatment	1	0.01	0.01	91.73	0.0000	***
poly(Plant_age_days, 3):Genotype	3	0.00	0.00	0.69	0.5586	
poly(Plant_age_days, 3):Treatment	3	0.00	0.00	10.79	0.0000	***
Genotype:Treatment	1	0.00	0.00	0.05	0.8168	
poly(Plant_age_days, 3):Genotype:Treatment	3	0.00	0.00	0.70	0.5503	
Residuals	274	0.03	0.00			

**Table A4:** Pod count analysis of deviance table.Significance (Sig.) indicated with stars; *** *p* value between 0 and 0.001, no stars *p* value > 0.1.

	Df	Deviance	Resid. Df	Resid. Dev	Pr (>Chi)	Sig.
NULL			51	179.07		
Genotype	1	0.79	50	178.28	0.3743	
Treatment	1	28.21	49	150.07	0.0000	***
Genotype:Treatment	1	2.16	48	147.91	0.1418	

**Table A5:** Bean weight ANOVA table. Significance (Sig.) indicated with stars; *** *p* value between 0 and 0.001, no stars *p* value > 0.1.

	Df	Sum Sq	Mean Sq	F Value	Pr (>F)	Sig.
Treatment	1	436.72	436.72	55.23	0.0000	***
Genotype	1	128.90	128.90	16.30	0.0002	***
Treatment:Genotype	1	101.49	101.49	12.83	0.0008	***
Residuals	48	379.58	7.91			

**Table A6:** Pollen viability ANOVA table.Significance (Sig.) indicated with stars; *** *p* value between 0 and 0.001, ** *p* value between 0.001 and 0.01, no stars *p* value > 0.1.

	Df	Sum Sq	Mean Sq	F Value	Pr (>F)	Sig.
Genotype	1	2192.92	2192.92	7.76	0.0085	**
Treatment	1	13,476.06	13,476.06	47.69	0.0000	***
Genotype:Treatment	1	2353.43	2353.43	8.33	0.0066	**
Residuals	36	10,172.45	282.57			
